# Adaptive regulation of the brain’s antioxidant defences by neurons and astrocytes

**DOI:** 10.1016/j.freeradbiomed.2016.06.027

**Published:** 2016-11

**Authors:** Paul S. Baxter, Giles E. Hardingham

**Affiliations:** School of Biomedical Sciences, University of Edinburgh, Edinburgh EH8 9XD, UK

## Abstract

The human brain generally remains structurally and functionally sound for many decades, despite the post-mitotic and non-regenerative nature of neurons. This is testament to the brain’s profound capacity for homeostasis: both neurons and glia have in-built mechanisms that enable them to mount adaptive or protective responses to potentially challenging situations, ensuring that cellular viability and functionality is maintained. The high and variable metabolic and mitochondrial activity of neurons places several demands on the brain, including the task of neutralizing the associated reactive oxygen species (ROS) produced, to limit the accumulation of oxidative damage. Astrocytes play a key role in providing antioxidant support to nearby neurons, and redox regulation of the astrocytic Nrf2 pathway represents a powerful homeostatic regulator of the large cohort of Nrf2-regulated antioxidant genes that they express. In contrast, the Nrf2 pathway is weak in neurons, robbing them of this particular homeostatic device. However, many neuronal antioxidant genes are controlled by synaptic activity, enabling activity-dependent increases in ROS production to be offset by enhanced antioxidant capacity of both glutathione and thioredoxin-peroxiredoxin systems. These distinct homeostatic mechanisms in neurons and astrocytes together combine to promote neuronal resistance to oxidative insults. Future investigations into signaling between distinct cell types within the neuro-glial unit are likely to uncover further mechanisms underlying redox homeostasis in the brain.

## Highlights

• Redox homeostasis is essential for brain health.
• Homeostasis involves interactions within the neurogliovascular unit.
• We describe cooperative contributions by neurons and astrocytes to redox homeostasis.

## 1 Introduction

The post-mitotic and non-regenerative nature of neurons means that either excessive oxidative damage needs to be avoided, or if possible reversed when it happens. Despite the well-documented increase in neuronal oxidative damage in the ageing brain, and the association of oxidative stress with several neurodegenerative disorders, the longevity of mammalian (particularly primate) neurons reflects successful redox homeostasis over many years. Understanding the basis for this illuminates a fundamental part of brain metabolism, points to key control nodes that may go wrong in disease, and also provides potential therapeutic targets for disorders associated with oxidative stress. The systems used inside the brain for neutralization of ROS or electrophilic xenobiotics are similar to those outside: the glutathione system, thioredoxin/peroxiredoxin system, superoxide dismutases, and catalase all play key roles in ROS neutralization and xeniobiotic adduction [1], and will be familiar to readers of this journal.

Many antioxidant genes in the systems outlined above are under the control of a master regulator of antioxidant defences, the transcription factor Nrf2, which binds to a promoter element called the antioxidant response element (ARE) present on these genes. Nrf2 is normally targeted for ubiquitin-mediated degradation by its endogenous inhibitor Keap1 [2], [3]. However, under conditions of redox imbalance, oxidative modification of Keap1 inhibits the Nrf2 degradation process, leading to Nrf2 accumulation in the nucleus, and the induction of ARE-containing genes [2], [3], [4]. By promoting ROS neutralization, xenobiotic clearance, and dampening inflammation, Nrf2 promotes cytoprotection in a variety of stress-related disorders affecting many tissues in the body, and is also anti-tumourigenic [3], [4]. There is also growing evidence that Nrf2 controls mitochondrial function directly by influencing fatty acid oxidation, respiratory substrate availability and ATP synthesis, as well as acting upstream to regulate mitochondrial ROS production [5], [6]. As such, the Keap1/Nrf2 system is an ideal homeostatic regulator of intrinsic cellular antioxidant defences and mitochondrial health. Moreover, the fact that Keap1-mediated Nrf2 degradation can be inhibited by a variety of electrophilic small molecules makes it a therapeutically attractive pathway for a variety of disorders [7]. Shortly after landmark discoveries by the Yamamoto laboratory regarding the role of Nrf2 and Keap1 in regulating ARE-containing genes [8], [9], interest began in understanding Nrf2 biology in the brain. As described below, this was driven by the laboratories of Jeff Johnson and Tim Murphy who found that Nrf2 had very different activities between neurons and astrocytes, and that Nrf2-mediated cytoprotective effects can be affected in a non cell-autonomous manner [10], [11]. This review will describe the very different roles of the Nrf2 pathway in neurons and astrocytes, and how Nrf2-dependent and -independent transcriptional programs in different cell types cooperate to promote redox homeostasis in the brain.

### 2 The Nrf2 pathway is weak in forebrain neurons

The first indication that the Nrf2-ARE pathway was particularly weak in neurons came from a study in neuronal and glial cells from the cerebellum [12]. It was observed that while the Keap1-inhibiting Nrf2 activator tBHQ induced ARE-containing genes GSTP1 and NQO1 in astrocytes, cerebellar granule neurons failed to respond. The authors also noted that basal expression of these genes was elevated in astrocytes compared to neurons [12], making it unlikely that the neuronal pathway was unresponsive due to being maximally active. Studies involving biolistic transfection of an ARE-reporter into coronal cortical slices also revealed highly preferential ARE reporter activity in astrocytes over neurons [13], and an astrocyte-focused response to both tBHQ and sulforaphane [11]. A potential explanation for lower neuronal Nrf2-ARE pathway activity was provided by the observation that cortical astrocytes express more Nrf2 protein basally than levels found in neuronally-enriched cultures [10]. The authors’ microarray study pointed to around 30-fold lower expression at the mRNA as well [10]. This difference was all the more remarkable given that the authors estimated that around 10% of neuronally-enriched cultures were astrocytes. Later qPCR studies by ourselves and others employing near-astrocyte free neuronal cultures corroborated this differential: cortical neurons were found to express approximately 100–1000-fold less Nrf2 than astrocytes [14], [15]. As if this were not enough to limit neuronal Nrf2 activity, neurons also have a greater capacity to promote degradation of what little Nrf2 is expressed, by possessing higher Cul3-dependent Nrf2 degradation capacity than astrocytes [15]. Consistent with all this, our lab observed no transcriptional response of neurons to either small molecule activators of Nrf2 (e.g. tBHQ) or to genetic activation of the pathway (Keap1 deficiency). The molecular basis for Nrf2 gene repression in neurons appears to be epigenetic in nature: neurons exhibit far lower levels of Nrf2 promoter histone H3 acetylation than astrocytes [14]. The process of Nrf2 gene repression takes place early in development: while Nrf2 expression and pathway activity are on a par with astrocytes at P0 in vivo and days-in-vitro (DIV) 2 in vitro, repression of expression, and reduction of promoter H3 acetylation has taken place by DIV 9 [14].

The biological reason why Nrf2 is expressed so weakly in maturing neurons may be that it promotes weaker antioxidant defences to facilitate redox signaling involved in neuronal development [16], [17]. Despite ectopic expression of Nrf2 in neurons being profoundly protective against oxidative insults [18], it retards structural and electrophysiological maturation [14]. Ectopic expression of Nrf2 appears to suppress the activity of developmentally important signaling pathways (JNK and Wnt) whose activity is promoted by redox signaling [19], [20], [21], [22]. Nrf2 activity restricts this signaling by providing a strong cellular redox buffer, preventing redox-dependent potentiation of these pathways [14]. In contrast, astrocytes evidently develop fine whilst expressing high levels of Nrf2, suggesting that the signaling pathways involved in their maturation (which remain incompletely understood [23]) may be less sensitive to redox status.

Despite the brake that Nrf2 puts on neuronal development, it remains an open question as to whether re-activation of the Nrf2 pathway once neurons have matured, may be a useful way of boosting neuronal antioxidant defences. De-repression of the Nrf2 promoter can be achieved partly by treatment with a histone deacetylase inhibitor [14], which, in combination with a Keap1-inhibiting Nrf2 activator, can modestly induce Nrf2 target genes in neurons [14]. Of note, enone-type electrophilic Keap1 inhibitors curcumin and NEPP11 are reported to induce Nrf2 target gene expression in neurons, unlike classical activators such as tBHQ [24], [25]. It is possible that these properties are due to curcumin’s capacity as an epigenetic regulator as well as a Keap1 inhibitor [26].

### 3 Weak Nrf2 activity in neurons means a reliance on astrocytes

Neurons in isolation have long been known to have limited intrinsic antioxidant defences: they express relatively low levels of catalase and do not contain high levels of GSH [1]. This is likely to be due in part to their very low basal Nrf2 pathway activity, since both catalase as well as key GSH biosynthetic and recycling enzyme genes are all controlled by Nrf2. Indeed, cortical neuronal expression of Cat and Gclc (Υ-glutamate-cysteine ligase catalytic subunit) are both far lower than that in astrocytes, and unlike astrocytes, expression levels are not reduced by Nrf2-deficiency [14]. Consistent with this, astrocytes are more resistant to oxidative insults than isolated neurons, and unlike neurons their vulnerability is increased by Nrf2-deficiency [14].

Neurons are metabolically highly active, and consume large amounts of ATP simply to maintain their resting membrane potential. Neurons have relatively low glycolytic capacity and limited scope to upregulate it upon increased energy demand [27]. As such, ATP production is met largely through mitochondrial oxidative phosphorylation [28], with astrocyte-derived lactate a potentially important source of oxidizable substrate [29]. A by-product of this dependency on oxidative phosphorylation however, is ROS generation. There is evidence that even in well-coupled mitochondria, ROS production does occur and correlates well with the rate of electron transfer through the various complexes [30], [31], [32], [33], [34]. Moreover, there is considerable evidence that elevated activity leads to an increase in mitochondrial metabolism [35] and increased mitochondrial ROS production, as well as ROS from non-mitochondrial sources such as activity-inducible enzymatic sources like NADPH oxidase [30], [36], [37], [38], [39], [40]. Thus, the high and variable metabolic in neurons would appear to indicate a high requirement for intrinsic antioxidant defences.

Nevertheless, despite their relatively low intrinsic antioxidant defences, it is self-evident that post-mitotic central neurons survive and are functional for many decades. The explanation for this apparent paradox is that neurons receive strong antioxidant support from surrounding glial cells, particularly astrocytes [1], [41]. Astrocytes have a high capacity for the production and storage of GSH, and release it into the extracellular medium in a manner that is increased in response to oxidative stress, via the transporter multidrug resistance protein 1 [1], [42]. This GSH is broken down extracellularly and cysteine-containing products taken up by neurons and used to synthesize their own GSH [1], [10], [41], [43]. Thus, astrocytes play a key role in providing to neurons the basic building blocks for GSH synthesis. Indeed, this whole process is controlled by Nrf2 and induction of it appears to be a major mechanism by which astrocytic Nrf2 activation protects nearby neurons, including human neurons [10], [41], [44], [45].

Moreover, since oxidative stress also induces Nrf2-dependent gene expression in astrocytes, including GSH pathway genes [46], [47], the capacity of astrocytes to provide neuronal support is also homeostatically regulated. Another signal that can activate astrocytic Nrf2 is chronic activation of astrocytic NMDA receptors, a Ca^2+^-permanent subtype of ionotropic glutamate receptor [15]. Application of NMDA directly to astrocytes triggers Cdk5-mediated Nrf2 phosphorylation and induction of Nrf2-dependent gene expression with the capacity to confer neuroprotection on nearby neurons [15]. Since both oxidative stress, and chronically elevated levels of ambient glutamate are both hall-marks of excitotoxic disorders such as ischemia, both pathways may be involved in the stress-induced activation of astrocytic Nrf2 that underlies some of the neuroprotective effects of ischemic preconditioning [46], [47], [48]. Of note, the developmental repression of Nrf2 in neurons roughly coincides with the onset of astrogliogenesis [14]. It is tempting to speculate that developing neurons require functional Nrf2-mediated antioxidant defences until there are sufficient astrocytes in the brain to provide external antioxidant support.

The demonstrably potent capacity of astrocytes to confer non cell-autonomous neuroprotection against oxidative insults points to the translational potential of small molecule Nrf2 activators. Indeed, there exists a wide range of these which have shown neuroprotective effects in variety of models of acute and chronic neurological disorders, including stroke, and Alzheimer’s, Parkinson’s and Huntington’s diseases, as well as MS [49], [50], [51]. Note that proof that the effects are mediated by astrocytes is currently lacking and would require (for example) trials in mice with a conditional deletion of Nrf2 in astrocytes.

Clear proof that astrocyte-driven Nrf2 activity is sufficient to confer neuroprotection in vivo has however been provided through the generation of transgenic mice over-expressing Nrf2 specifically in astrocytes, and tested against models of a variety of neurological and neurodegenerative disorders including Parkinson’s disease, Huntington’s disease, Alexander disease and ALS [50], [52], [53], [54], [55]. It is worth noting that the exact mechanism of neuroprotection is unclear in these models. While it could be the aforementioned astrocyte-derived GSH release, other potential mechanisms also exist, such as maintenance of astrocyte health and function, or preventing reactive astrogliogenesis. Nevertheless, targeting Nrf2 offers a promising therapeutic strategy against neurodegenerative disorders associated with oxidative stress [51], and potentially psychiatric ones too [56]. Such an approach of targeting endogenous gene expression programs is conceptually different to classical small molecule antioxidant therapies which have met with limited success in the clinic [57].

### 4 Dynamic regulation of neurons’ own antioxidant defences

Although the astrocytic supply of precursors for GSH synthesis to neurons represents a key factor in enabling neurons to neutralize ROS, it is not the only determinant. Neurons must be able to use these precursors to synthesize GSH, as well as possess the systems to utilize and recycle GSH, as well express other important intrinsic antioxidant systems such as those based around thioredoxin and peroxiredoxins [58], [59]. For example, in neurons where the GSH biosynthetic enzyme gene Gclc was knocked down, astrocyte-derived GSH precursors are unable to confer their normal protection [60]. Both the catalytic (Gclc) and modifier (Gclm) genes of GCL are Nrf2 target genes, as are other genes in the GSH system such as glutathione reductase, and several glutathione peroxidases [3]. However, since neurons do not possess robust levels of Nrf2 they lack the ability to homeostatically regulate GSH system gene expression this pathway via oxidative stress-induced Nrf2 activation.

Interestingly, however, many GSH pathway genes and other known Nrf2-regulated antioxidant genes such as Gclc, Gsr, Srxn1 and xCT are dynamically regulated in neurons by synaptic activity [61], [62], [63]. At face value this makes sense, since the process of synaptic activity and action potential firing is energetically expensive, placing further ATP demands on the neuron which is met by increased metabolic activity [64], [65] and associated increased ROS production [30], [36], [37], [38], [39]. Consistent with this, increased synaptic activity causes an immediate increase in GSH utilization [66], unsustainable unless production can be increased to counter-balance this increased demand. By inducing key GSH system genes, synaptic activity increases the capacity of neurons to synthesize, utilize and recycle GSH [62]. Thus, it could be that the coordinated control of antioxidant genes by synaptic activity represents a homeostatic control that helps to match intrinsic antioxidant defence system capacity to the demands of an active neuron. Mechanistically, neuronal activity regulates these known Nrf2 target genes independent of Nrf2 [67], targeting activity-responsive transcription factors that also regulate these genes which include ATF4 in the case of xCT [63], and AP-1 in the case of Srxn1 [61]. Indeed, an activity-responsive AP-1 binding site is actually embedded within the functional ARE of the Srxn1 promoter [68], something that is observed in a variety of Nrf2 target genes [69]. Ca2+ signaling, particularly through the NMDA receptor subtype of ionotropic glutamate receptors, is a major mediator of activity-dependent regulation of these antioxidant genes [61], [62]. Indeed, NMDA receptor blockade in vivo causes a reduction in brain Gclc expression, GCL activity and GSH levels [62]. Moreover, this blockade is associated with neurodegeneration which can be ameliorated by supplying the brain with a cell-permeable form of gamma-glutamyl-cysteine, the product of GCL catalysis [62]. Thus, in the absence of a significantly activatable Nrf2 pathway, the activity-dependent regulation of antioxidant genes may represent an important homeostatic regulator of a neuron’s intrinsic antioxidant systems. Conceptually, this form of activity-dependent homeostasis draws parallels with others, such as homeostatic plasticity of synaptic strength, intrinsic excitability or circuit structure [70], [71], [72].

### 5 Neuron-astrocyte cooperation to promote neuroprotection

**Fig. 1:**
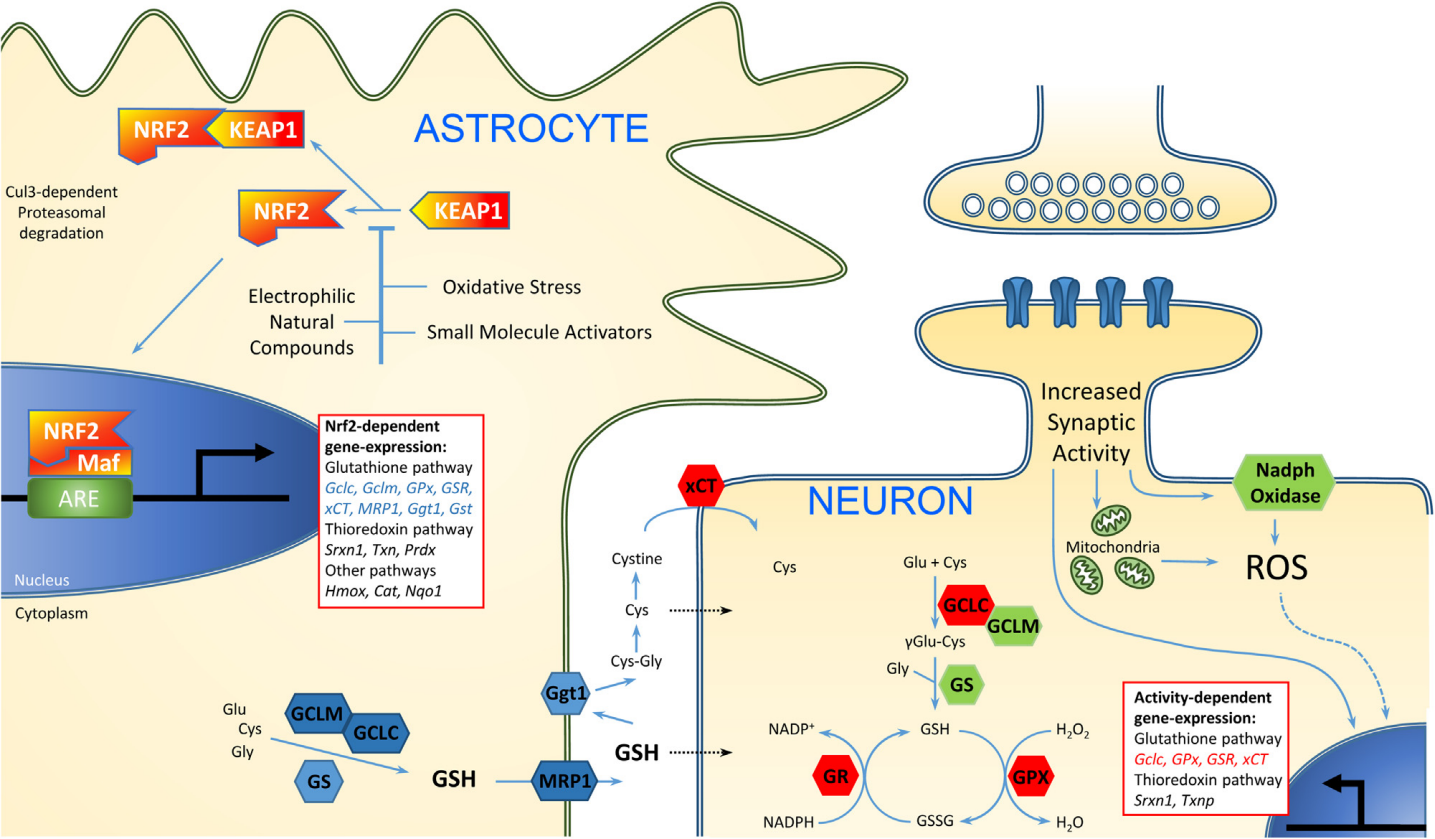
Astrocytes and neuronal activity play distinct, cooperative roles in neuronal redox homeostasis. Astrocytes respond to mild oxidative stress and other inducers of the Nrf2 pathway by turning on a program of Nrf2-mediated antioxidant gene expression, with consequent synthesis of GSH. GSH is exported via Mrp1 and degraded (by Ggt1), with one or more degradation products taken up by neurons and fed into their own GSH biosynthesis pathway. Thus, while astrocytes provided support in providing the building blocks for GSH production, neurons still require the capacity to make use of these raw materials. This capacity is controlled by synaptic activity-induced signaling pathways, via the transcriptional control of a number of key genes involved in GSH synthesis, peroxidation and recycling.

A consequence of neuronal antioxidant genes being activity-responsive is that synaptic activity can increase the capacity of neurons to utilize the support provided by astrocytes. In other words, while astrocytes are an important source of cysteine-containing precursors that neurons take up and use to make their own GSH, by increasing the GSH biosynthetic capacity of neurons, neuronal activity makes them better able to use these astrocyte cysteine-containing precursors to boost their intrinsic defences. As described above, astrocytic support is under control of the Nrf2 pathway. Thus, while the activation of astrocytic Nrf2, and the induction of synaptic activity, are separately neuroprotective, combined they have an additive effect on the resistance of neurons to oxidative insults [62]. The cooperative nature of the interaction is depicted in Fig. 1.

One outstanding question, however, surrounds whether the influence of synaptic activity on antioxidant gene expression is limited to neurons. As noted above, exogenous activation of astrocytic NMDA receptors can activate Nrf2, raising the possibility that neuronal glutamate release could control astrocytic Nrf2 [15]. Indeed, a recent study reported that stimuli that promote synaptic activity, or neuronal depolarization, increase nuclear accumulation of Nrf2 in astrocytes, raising the possibility that synaptic activity controls the astrocytic Nrf2 pathway [73]. The consequences of this for astrocytic Nrf2 target gene induction were not entirely clear in the study, however, because the authors studied expression of these genes in a mixed population of neurons and astrocytes, meaning that the cell-type where they were being induced was unclear. This is particularly relevant because (as stated above) neuronal activity also induces known Nrf2 target genes in the neurons themselves, independently of Nrf2 [67]. While we have not observed astrocytic Nrf2 increases in response to neuronal activity [67], we cannot rule out the possibility that it does happen under certain situations. Moreover, the study by Habas et al. [73] underlines the fact that the general issue of astrocytic gene regulation by neuronal synaptic activity is one that requires further investigation. While the mechanisms and consequences of activity-dependent gene expression in neurons have been the topic of hundreds of papers over the past 30 years [74], [75], little is known about whether, or to what extent, neuronal activity influences the transcriptome of surrounding glial cells, including astrocytes. Given the intimate metabolic and functional coupling between neurons and astrocytes [28], a clearer understanding of the mechanisms and consequences of reciprocal signaling between these two cell types is needed. For studying gene regulation, this will require neuronal activity to be induced in a mixed neuronal/astrocytic co-culture, followed by separation techniques that allow the two transcriptomes to be distinguished. Unfortunately, physical separation prior to stimulation (e.g. astrocytes and neurons separated using trans-well inserts) is not ideal because astrocytic morphology and function in the absence of neurons is different to that found in vivo [23], [76]. More broadly, a greater understanding of reciprocal interactions between all cell types within the neuro-glial unit is required to gain a full picture of brain redox homeostasis. ROS do not simply damage neurons, but disrupt white matter integrity, as well as push microglia towards pro-inflammatory phenotypes. Indeed, Nrf2 mRNA expression in microglia is even higher than in astrocytes [77], pointing to an important regulatory role in this cell type too. The coming years will likely uncover new homeostatic mechanisms the help maintain the brain’s redox balance over a lifetime.

## Figures and Tables

This increased capacity may be a homeostatic response, since synaptic activity is metabolically expensive and can lead to increased ROS production both from mitochondrial sources and Ca^2+^ dependent NADPH oxidase.
